# Cognitive performance and mood after a normal night of drinking: A naturalistic alcohol hangover study in a non-student sample

**DOI:** 10.1016/j.abrep.2019.100197

**Published:** 2019-06-15

**Authors:** Lydia E. Devenney, Kieran B. Coyle, Joris C. Verster

**Affiliations:** a School of Psychology, Life and Health sciences, Ulster University, BT52 1SA Londonderry, Northern Ireland; b Division of Pharmacology, Utrecht University, Utrecht, the Netherlands; c Centre for Human Psychopharmacology, Swinburne University, Melbourne, Australia

**Keywords:** Alcohol, Hangover, Cognitive functioning, Mood, Non-student sample

## Abstract

**Aims:**

The alcohol hangover is typically investigated in student samples. However, alcohol hangovers are also reported by non-student drinkers, beyond the age and drinking behaviors of a student sample. The aim of this study was to investigate the effects of a normal night of alcohol consumption on next-day cognitive performance in a non-student sample.

**Methods:**

Participants (*N* = 45) were recruited from a public drinking setting and participated in a naturalistic study comprising of a hangover test day and alcohol-free control day. On each test day, mood and hangover severity were assessed and participants completed a cognitive test battery consisting of a Stroop test, Eriksen's flanker test, spatial working memory test, free recall test, choice reaction time test, and intra-extra dimensional set shifting test.

**Results:**

On the hangover day, significantly impaired performance was revealed on all tests, except the intra-extra dimensional set shifting test. On the hangover day, significantly lower mood scores were observed for alertness and tranquility.

**Conclusion:**

The current study in a non-student sample confirms previous findings in student samples that cognitive functioning and mood are significantly impaired during alcohol hangover.

## Highlights

• The alcohol hangover is typically investigated in student samples.
• Alcohol hangovers are also reported by non-student drinkers.
• On the hangover day, significantly impaired performance was revealed on cognitive functioning and mood.
• In both student and non-student samples, comparable impairments are found during alcohol hangover.

The alcohol hangover is typically investigated in student samples. Alcohol hangovers are also reported by non-student drinkers. On the hangover day, significantly impaired performance was revealed on cognitive functioning and mood. This study confirmed that in both student and non-student samples, comparable impairments are found during alcohol hangover.

## 1 Introduction

An alcohol hangover is defined as the combination of mental and physical symptoms that are experienced the day after an episode of heavy alcohol consumption, starting when blood alcohol concentration (BAC) approaches zero (Van Schrojenstein Lantman, van de Loo, Mackus, & Verster, 2016). Various symptoms and complaints can be present during the hangover state (Penning, McKinney, & Verster, 2012), and these may differ in severity, in both between and within participants designs. Most commonly reported complaints, reported by >90% of drinkers, are fatigue, thirst, concentration problems, and headache (Van Schrojenstein Lantman, Mackus, van de Loo, & Verster, 2017). In addition, it was shown that hangover symptoms may have a differential impact on mood, cognitive functioning and physical performance (Van Schrojenstein Lantman, Mackus, van de Loo, & Verster, 2017). Together, these effects may significantly impair daily activities such as driving a car (Verster et al., 2014, Verster, Van Der Maarel, McKinney, Olivier, & De Haan, 2014).

Studies examining cognitive functioning during an alcohol hangover have revealed inconclusive results. That is, whereas most findings suggest that cognitive performance is significantly impaired the day following a night's drinking (e.g., Anderson, 1999; Grange, Stephens, Jones, & Owen, 2016; Kim, Yoon, Lee, Choi, & Go, 2003; McKinney & Coyle, 2004; McKinney, Coyle, Penning, & Verster, 2012; Roehrs, Yoon, & Roth, 1991; Rohsenow et al., 2010) other studies did not find significant performance impairment (e.g., Carroll, Ashe, & Roberts, 1964; Myrsten, Kelly, Neri, & Rydberg, 1970; Colins & Chiles, 1980; Chait & Perry, 1994; Finnegan, Hammersley & Cooper, 1998; Verster, Van Duin, Volkerts, Schreuder, & Verbaten, 2003; Howland et al., 2010). There are several methodological differences between the studies and many have methodological shortcomings that may account for the observed differences in outcomes. One of these may be the composition of the sample under investigation.

Most scientific studies investigating the alcohol hangover are conducted in student samples or comparable young adults. Likewise, most knowledge of mood effects and cognitive and psychomotor functioning during the hangover state comes from these young samples (e.g. Anderson, 1999; Collins, Bowman, & Sutherland, 1971; Hogewoning et al., 2016; Howland et al., 2010; Laurell & Törnros, 1983; McKinney & Coyle, 2004). Studies conducted in non-student samples were often male-only samples (e.g., Roehrs et al., 1991; Lemon, Chesher, Fox, Greeley, & Nabke, 1993; Streufert et al., 1995, Finnigan et al., 1998b). It can be questioned to what extent this data is representative of adult social drinkers and non-student samples, because research suggested that drinking behaviors differ between student and non-student samples (Gill, 2002; Kypri, Cronin, & Wright, 2005; Wilsnack, Vogeltanz, Wilsnack, & Harris, 2000). Similarly, a variation in drinking behaviors across ages has been demonstrated using several cohorts containing longitudinal data. In adolescents, drinking often begins at under 10 units per week and increases in males to 20 units per week at the age of 25. A similar trajectory was also found for female respondents but with a considerably lower peak of 7 to 8 units (Britton, Ben-Shlomo, Benzeval, Kuh, & Bell, 2015). Alcohol consumption then declines and stabilizes during middle age. There is also considerable evidence to showing age-related changes in cognition as well as mood regulation. Finally, evidence of changes in hangover symptom severity with age have also been demonstrated (Fozard, Vercruyssen, Reynolds, Hancock, & Quilter, 1994; Murman, 2015; Soubelet & Salthouse, 2011; Tolstrup, Stephens, & Grønbæk, 2014).

A reason for using student samples is that they are easy to recruit at the universities where investigators conduct their research. This limitation is not unique to hangover research but seen across the field of psychological research (Hanel & Vione, 2016).

Whereas student populations are usually recruited at universities, suitable non-student participants of hangover studies might be found at local pubs, including public houses. With this in mind, a public house was chosen as an appropriate venue for recruiting a non-student sample in the current study. A naturalistic study design was applied, to mimic real life alcohol consumption levels and drinking behaviors. As a result, researchers did not interfere with the (drinking) activities of participants. Participants' cognitive functioning and mood was assessed both the day after drinking alcohol, and on an alcohol-free control day.

In the interest of repetition, Eriksen's Flanker and the Stoop task were chosen to measure selective attention (Stephens, Grange, Jones, & Owen, 2014). These tasks have traditionally shown sensitivity to a hangover, and thus have become somewhat indicative of the presence of a hangover. Inconsistencies in findings relating to working memory exists in hangover research, therefore repetition of a free recall task using the same stimuli as McKinney and Coyle (2004) in a non-student sample was of interest. The CANTAB has been widely used to investigate mood and drug related cognitive impairment (Arvind et al., 2017; Bø, Billieux, Gjerde, Eilertsen, & Landrø, 2017; Schuster et al., 2018). Tasks from the CANTAB were chosen in order to gain specific insight into aspects of cognition that are not widely explored during a hangover e.g. rule acquisition (attentional set-shifting).

Taken together, there is a lack of data on alcohol hangover effects in non-student populations. Therefore, the aim of this study was to recruit adult social drinkers from their local public house and examine their cognitive functioning the day following a normal night of alcohol consumption and compare it with their performance after an alcohol-free evening.

## 2 Methods

### 2.1 Participants and design

*N* = 45 social drinkers (*N* = 25 men and *N* = 20 women) were recruited to participate in a naturalistic study on the effects of a night's drinking on cognitive performance at a public house in Ireland (town population, 6839; Census, 2011). Recruitment and participation took place from the opening of the premises (between 10.30 a.m. and 12.30.p.m) until 3 p.m.

The study followed a repeated measures design using a within participants variable of state (hangover /no hangover). The dependent variables were derived from the measures of cognitive performance and the responses to the subjective questionnaires. Participants were allocated to order (order 1 = hangover/no hangover, *n* = 26; order 2 = no hangover/hangover, *n* = 19) depending on alcohol consumption on the night before recruitment. For example, those that consumed alcohol the evening before recruitment were allocated to order 1 and those that did not were allocated to order 2. Participants were recruited in the public house on a voluntary basis and no incentive was offered for participation in the study. The frequency of attendance on both drinking and non-drinking days by patrons enabled hungover and non-hungover data (both testing sessions) to be collected within a 5–10 day time frame. For example, on the day following a drinking occasion, patrons would often socialize in the public house without consuming alcohol. In this way, the investigators did not interfere with the alcohol consumed by participants or their accompanying behaviors. Participants with a BAC level above zero were excluded from participation. BAC was assessed using a Lion Alcolmeter sd-400 breathalyzer. In addition, data from those that scored zero on the Acute Hangover Scale (AHS) were not included in the analysis (*N* = 2). Three participants were excluded because they consumed drugs (cannabis, N = 2; ecstasy = 1) on the day of alcohol consumption, and two other participants were excluded for this reason on the control day (cannabis, N = 2). Although 51.1% of the sample identified as past / current drug consumers, these participants were not removed from the study as they tested negative on the test days. Written informed consent was obtained from all participants. Ethical approval for this study was obtained from the ethics committee at Ulster University.

### 2.2 Procedure

Testing took place in an office above a public house in the local area and lasted between 45 and 60 min. Participants completed the Acute Hangover Scale (Rohsenow et al., 2007), demographic information was collected, and information was gathered about the number of hours sleep and previous night's alcohol consumption. Thereafter, Herbert, Johns, and Doré (1976) adaption of the Bond and Lader mood scale was completed. Following this, a series of cognitive tests were administered. Of note, order of task administration was reversed for half of the participants.

### 2.3 Eriksen's Flanker Task

**Fig. 1:**
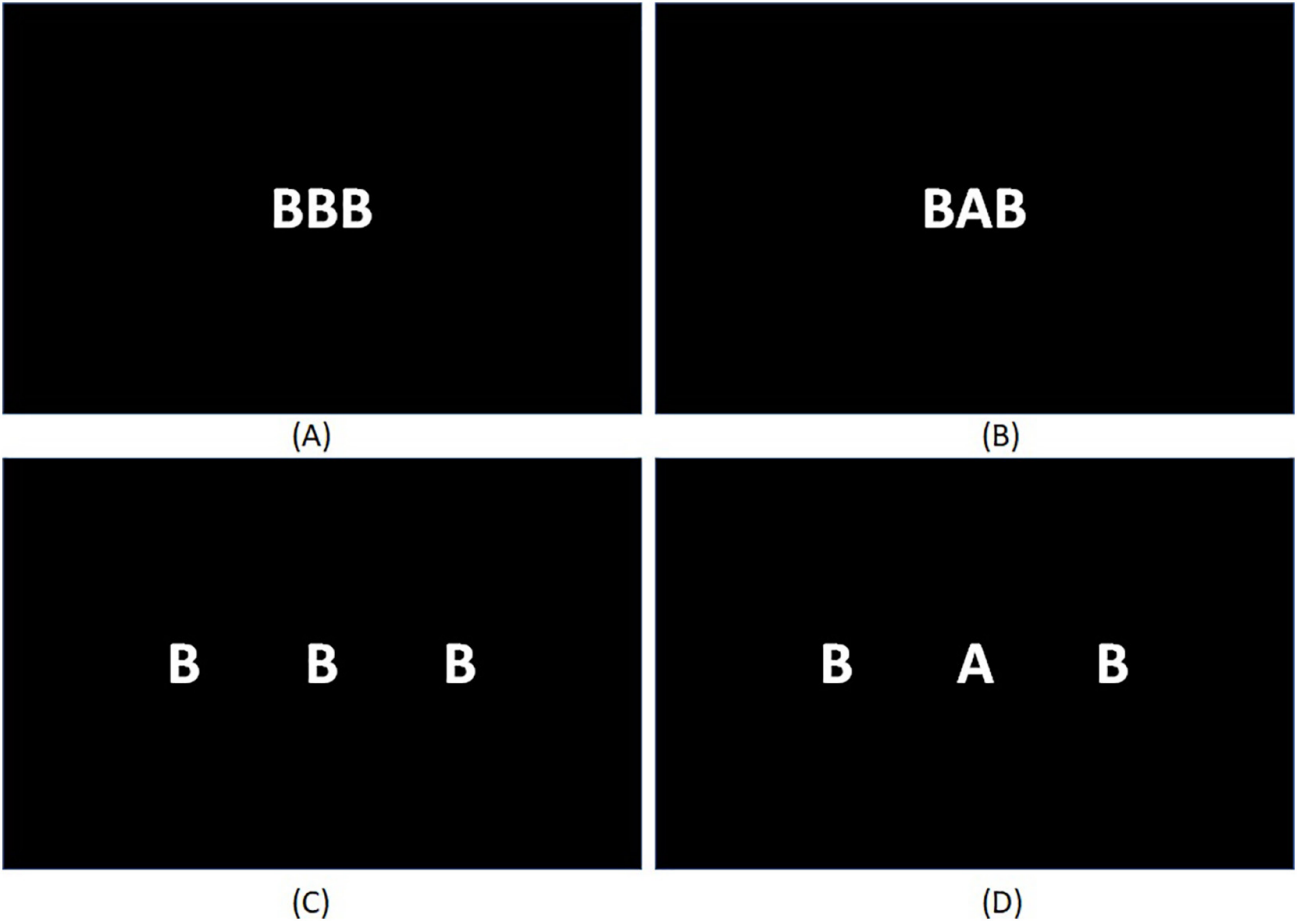
The Eriksen's flanker task. Participants are instructed to respond by button press to the middle target letter. Distractor letters on the left or right can either be compatible (same letter) or incompatible (different letter). In addition they can be depicted on a near or far distance from the target letter. Shown are examples of (a) compatible distractors and near distance, (b) incompatible distractors and near distance, (c) compatible distractors and far distance, and (d) incompatible distractors and far distance.

In this selective spatial attention task the targets and distracters consist of the letters A and B (Eriksen & Eriksen, 1974). Distracters are presented at either side of the target and appear either near (1 cm) or far (3.4 cm) from the target. Distracters are either compatible (A-A-A) or incompatible with the target (B-A—B) (See Fig. 1). Once instructions are read, participants are required to complete a practice block and eight testing blocks with eight trials in each, including equal proportions of stimuli distractor types.

Letters are presented for 1000 ms and cues for 500 ms. The cues were three asterisk symbols proportionately spaced across the center of the screen. In all instances, font was set at size 52 Times New Roman in Black. Participants are required to respond to the target letter by pressing an appropriate key as quickly and accurately as possible. Task variables include ‘total errors’, ‘distance’ and ‘compatibility’ response times. A distance variable is calculated by subtracting the reaction times to stimuli with near distractors from reaction times to stimuli with distractors that are far away. Similarly, a compatibility variable is calculated by subtracting compatible distractor type response times from incompatible distractor type responses.

### 2.4 Stroop test

**Fig. 2:**
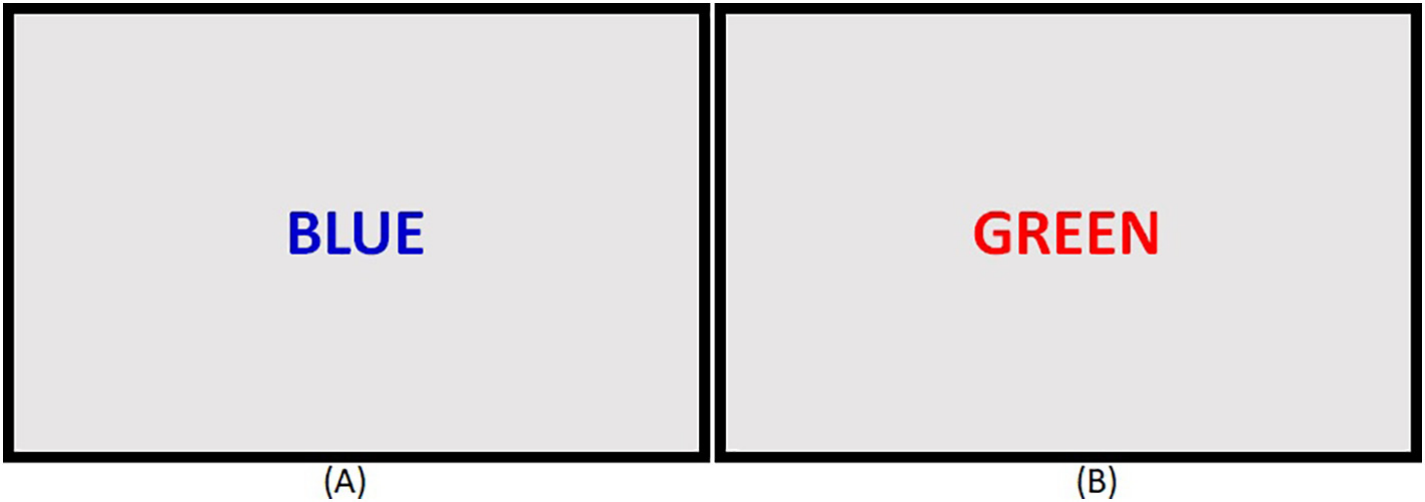
Stroop test. Participants are instructed ignore the text-meaning of the words, and required to respond by button press to the font color only. The presented items can be (a) congruent (‘blue’ printed in ‘blue color’), or (b) incongruent (‘green’ printed in ‘red color’). Usually, when word meaning and printed color are incompatible, i.e. the process called interference, response times will be prolonged (Stroop, 1935). Subjects are instructed to respond as quickly and accurate as possible. Each stimulus remains on the screen until a response is made. An interval ‘+’ appears for 1000 ms between stimuli. (For interpretation of the references to color in this figure legend, the reader is referred to the web version of this article.)

In this task, words are presented on the screen one at a time in Blue, Green, Red, Purple and Brown as used in the original task (Stroop, 1935). Ignoring the text-meaning of the words, participants are required to respond to the font color only by using the corresponding buttons on the keyboard provided (See Fig. 2).

This task contains one practice block and 5 testing blocks, each containing 10 trials. Congruent items are presented on 1/3 of the trials and incongruent items are presented in 2/3 of the randomized trials. Dependent variables include the number of errors, congruent and incongruent items, and Stroop interference. Stroop interference represents the difference between RTs for correct congruent (e.g. red presented in red font) and correct incongruent items (e.g. red presented in green font). Only correct responses were included in the Stroop interference calculation.

### 2.5 Free recall

The free recall task consists of twenty words that are presented in uppercase lettering on a computer screen at a rate of one word every two seconds. The words were selected from the handbook of Semantic Word Norms (Toglia & Battig, 1978) and have been used previously in hangover research by McKinney et al. (2012). In the minute directly following presentation participants are required to write down as many words as they can remember. The dependent measure is the number of correctly recalled words.

### 2.6 CANTAB - spatial working memory test

**Fig. 3:**
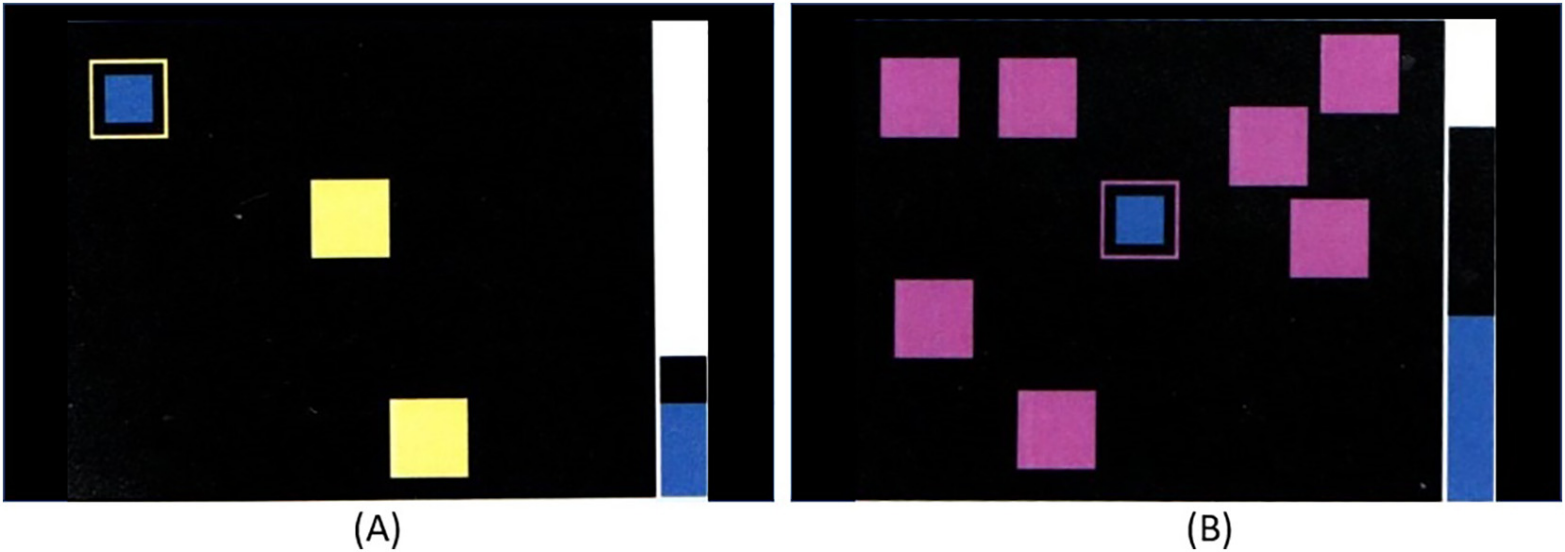
The CANTAB spatial working memory test. Fig. 3 (a) shows a sample from Block 1 (3 boxes). By process of elimination, participants must find the token from within one of the 3 boxes. The top left corner of the display demonstrates a blue token within a yellow box, a token will not be found in this box again. Fig. 3 (b) refers to a trial from the final block (8 boxes) within the test. Here the participants must find the token from a greater number of possible locations. It can be seen from the column to the right of the display, that half of the tokens have been located. © Copyright 2018 Cambridge Cognition Limited. All rights reserved. (For interpretation of the references to color in this figure legend, the reader is referred to the web version of this article.)

The CANTAB spatial working memory task requires retention and manipulation of visuospatial information. This task tests executive functioning and as well as one's ability to manipulate remembered items (Cambridge Cognition, 2018a). As a result, this task represents a novel approach to memory measurements as previous research on memory during a hangover have applied immediate and delayed recall tasks (McKinney & Coyle, 2007; Taylor, Dolhert, Friedman, Mumenthaler, & Yesavage, 1996). In this task, participants must touch the colored squares in order to find a blue token (See Fig. 3). A number of colored boxes are shown on a black screen, and the subject is tasked with finding a yellow ‘token’ (smaller box) which can be found inside the boxes presented on the screen. Once found, the tokens are automatically used to fill up an empty column on the right-hand side of the screen. Of note, when a token is found within a particular box, it will *never* be found in that box again, once the column is filled, a new block begins. Task difficulty varies as the number of boxes from which to look inside increases from 3 to 8, and the color and position of the boxes changes from trial to prevent predictability. The most efficient strategy is to choose an order to press (open) the boxes, and start over in the same order each time a blue token is found. Outcome measures include number of errors (selecting boxes that have already been visited) and strategy. Strategy represents the number of times that a participant begins a new trial with a different box. Higher strategy scores indicate poorer use of the best strategy.

### 2.7 CANTAB - intra-extra dimensional set sifting test

**Fig. 4:**
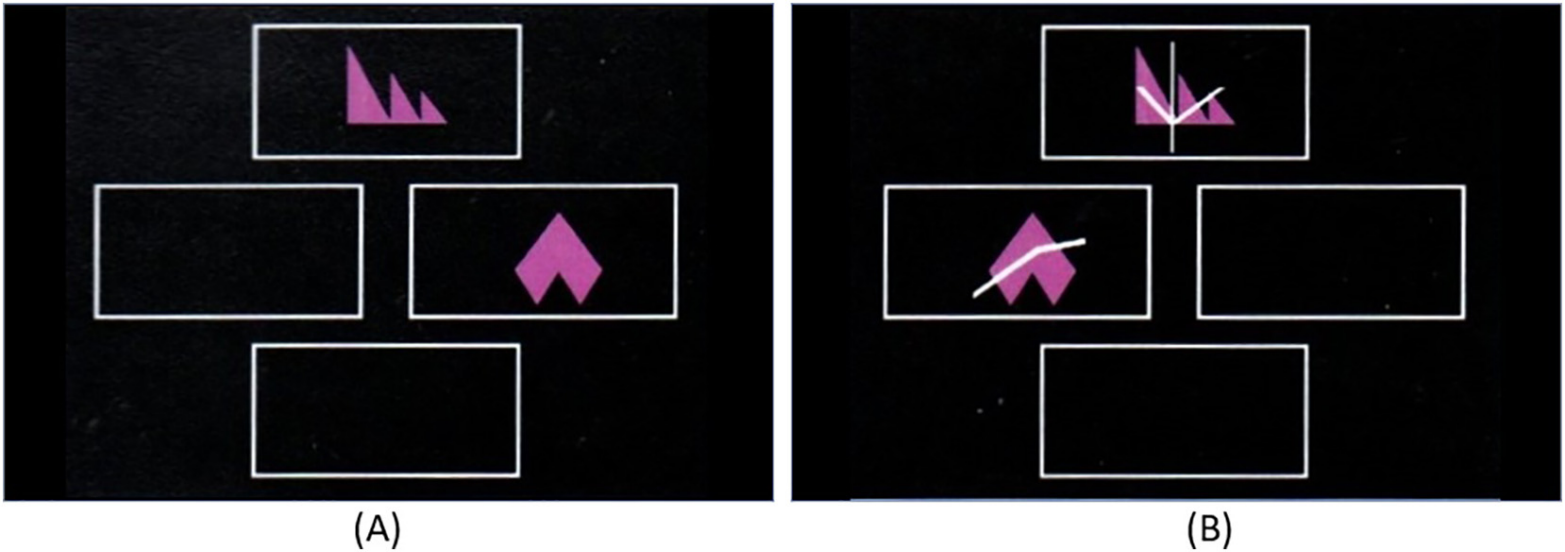
The CANTAB intra-extra dimensional set sifting test. Fig. 4 (a) shows an intra dimensional trial (Block 1). Here the shape of the pink objects determines the correct response. Fig. 4 (b) shows an extra dimensional trial from which the white lines determine the correct response (Block 8). © Copyright 2018 Cambridge Cognition Limited. All rights reserved. (For interpretation of the references to color in this figure legend, the reader is referred to the web version of this article.)

This test is a computerized analogue of the Wisconsin card sorting task which features visual discrimination and attentional set formation maintenance, shifting and flexibility of attention (Cambridge cognition, 2018b; Rock, Roiser, Riedel, & Blackwell, 2014; Rogers et al., 1999). In this task, participants must use feedback (correct, incorrect) to work out a rule that determines which stimulus (box) is correct. The task starts with four large boxes, two of these include individually shown white lines or pink shapes corresponding to intra-dimensional shifts in rules. In all cases, participants are required to choose one of two options (two boxes are empty in all trials), once identified, subject must continue to select the correct box (See Fig. 4a). After six correct responses, the stimuli and/or rule changes. Gradually, the task becomes more complex (e.g., white lines overlaid on pink shapes within two boxes) also requiring extra-dimensional rule shifting (Fig. 4b). In these complex cases, participants must identify the correct box which contains two items (pink shapes *and* white lines) rather than one. Outcome measures are the number of intra- and extradimensional errors (i.e., failing to identify the strategy within 6 trials).

### 2.8 CANTAB - choice reaction time test

**Fig. 5:**
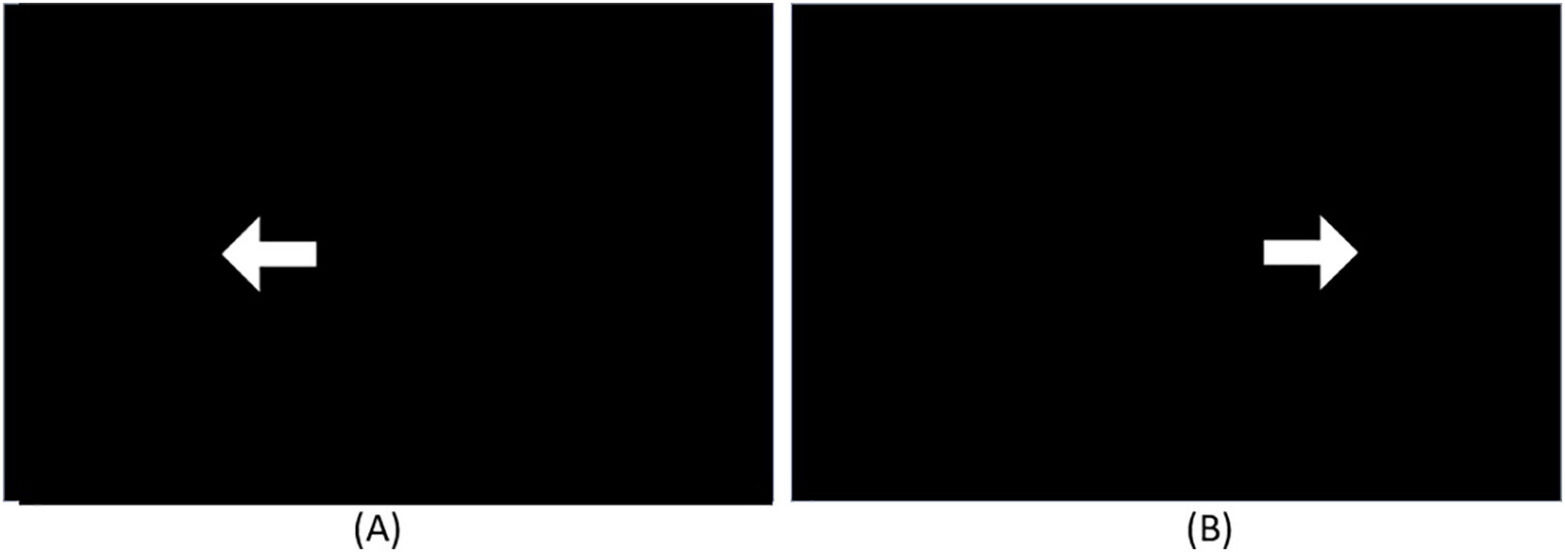
The CANTAB choice reaction time test. Participants are instructed to respond as quickly and accurate as possible to arrows facing (a) left and (b) right. © Copyright 2018 Cambridge Cognition Limited. All rights reserved.

The choice reaction time task measures alertness and psychomotor skills (Cambridge Cognition, 2018c). Speed and accuracy on this task reflect the rates of information processing. The task requires participants to respond to two possible stimuli using a touchpad. The stimuli are an arrow facing right and an arrow facing left (See Fig. 5). Participants are asked to respond accordingly using the right and left buzzers. After a response, participants are notified if their responses were correct/incorrect. The task takes approximately 7 min to complete and mean latency (RT) and number of errors were the outcome measures.

### 2.9 Herbert, Johns and Doré mood scale

Each participant was given questionnaires to complete before beginning the cognitive tasks. Mood was measured using an 18 item bi polar visual analog scale developed by Bond and Lader (1981) and adapted by Herbert et al. (1976). The scale was administered immediately before objective measures were carried out in order to gain an accurate record of mood at the time of testing. The questionnaire contained the following items that were presented at opposite ends of an 8 cm line: alert/drowsy, contented/discontented, calm/excited, troubled/tranquil, strong/feeble, mentally slow/quick witted, muzzy/clear headed, tense/relaxed, incompetent/proficient, happy/sad, antagonistic/friendly, interested/bored, withdrawn/sociable, depressed/elated, self-centered/outward going, well-coordinated/clumsy, and lethargic/energetic. Participants were required to place a mark on the line at a position which indicated how they were currently feeling. The raw scores for each line of bipolar items were then derived from the distance of the mark from the item on the left. In addition, the data were collapsed into two (sum score) variables: alertness and tranquility.

### 2.10 Statistical analysis

Statistical analysis was conducted with SPSS, version 24. Mean (SD) was computed for each variable. Participants with an AHS score of zero were excluded from the analysis, as this indicates the absence of having an alcohol hangover. Response time data above 2500 ms in the Stroop task were excluded from the study (Wykes, 1996) and a cut-off was set at 1500 ms for Eriksen's Flanker task (White, 2010). Of note, only correct responses were analyzed for response time measures. Results from the hangover day and control day were compared using a paired *t*-test analysis except for analysis on Stroop interferences and Eriksen's Flanker task, in which cases two way (congruency x state) and three way (distance x compatibility x state) repeated measures analyses were applied respectively. Differences were considered significant if *p* < .05. A Bonferroni correction as applied to account for multiple comparisons in the mood scale (statistical significance level *p* < .003).

## 3 Results

Forty-five participants participated in the study. Excluding those with AHS = 0, data from *N* = 43 participants was analyzed (23 male, 20 female). Their demographics are summarized in Table 1. Their mean (SD) age was 31.4 (11.7) years old, within an age range of 19–60 years old. On the night before the hangover test day participants consumed a mean (SD) of 15.4 (9.4) UK units of alcohol (1 unit = 8 g of alcohol). Of note, a t-test was carried out to investigate the differences in sleep across hangover and control days, the results did not reach significance (t(42) = 0.73, *p* = .47).

**Table 1:** Demographics and alcohol consumption characteristics of the sample. Significant differences between men and women (*p* < .05) are indicated by *.

	Overall	Men	Women	*p*-value
N	43	23	20	
Age (years)	31.4 (11.7)	32.0 (12.8)	30.8 (10.4)	0.74
Age of first alcoholic drink	15.1 (2.5)	14.3 (2.5)	16.0 (2.2)	0.02*
Hours of sleep (Hangover day)	7.5 (1.4)	7.5 (1.3)	7.4 (1.5)	0.94
Hours of sleep (control day)	7.7 (1.6)	7.7 (1.4)	7.7 (1.8)	0.88
Alcohol consumed on night	15.4 (9.4)	17.8 (11.4)	12.5 (5.5)	0.07
Alcohol hangover severity	15.0 (9.9)	14.0 (10.4)	16.3(9.5)	0.47

For the cognitive tests and mood assessments, the analysis revealed no significant interactions between State and Order, or Gender and Order. Therefore, the following sections describe direct comparisons between the hangover and control day only. Table 2 summarizes the results of cognitive test performance.

**Table 2:** Summary of the results from the cognitive test battery. Significant differences between the hangover and control day (p < .05) are indicated by *.

	Hangover day	Control day	Cohen's d	p-Value
Stroop test				
Congruent (ms)	1250.2 (255.5)	1152.2 (284.7)	0.31	0.05*
Incongruent (ms)	1588.6 (351.3)	1447.0 (351.5)	0.35	0.03*
Interference/congruency (ms)	337.1 (192.1)	294.3 (181.7)	0.19	0.22
Number of errors	5.4 (1.8)	5.5 (1.8)	0.04	0.81

Eriksen's Flanker test				
Overall	548.4 (116.2)	511.9 (67.8)	0.28	0.04*
Compatibility (dif.)	58.3 (89.8)	57.9 (58.5)	0.00	0.83
Distance (dif.)	62.4 (84.5)	68.9 (61.7)	0.06	0.65
Number of errors	2.4 (1.9)	3.0 (4.2)	0.10	0.50

CANTAB-intra-extra dimensional set shifting test				
Extra-dimensional errors	13.6 (18.4)	9.7 (16.5)	0.24	0.12
Intra-dimensional errors	6.6 (5.5)	5.6 (2.7)	0.17	0.27

CANTAB-Spatial working memory test				
Number of errors	20.6 (18.0)	15.0 (13.7)	0.35	0.03*
Strategy score	29.8 (6.9)	27.5 (6.8)	0.30	0.06
*Free recall test*				
Primacy	2.9 (1.3)	3.5 (1.3)	0.29	0.06
Intermediate	2.2 (1.7)	2.7 (1.5)	0.24	0.11
Recency	2.3 (1.3)	3.1 (1.5)	0.44	<0.001*
Total words recalled	7.5 (3.1)	9.5 (2.7)	0.60	<0.001*

CANTAB–choice reaction time test				
Latency	321.9 (41.0)	301.4 (26.5)	0.61	<0.001*
Number of errors	0.4 (0.6)	0.5 (0.9)	0.13	0.70

A main effect of day was found for incongruent Stroop response times (t(42), 2.32, *p* = .03) and congruent Stroop response times (t(42), 2.04, *p* = .05). In relation to repeated measures analysis of Stroop performance, a main effect of state (F(1, 42) = 5.28, p = .03) and congruency (F(1, 42) = 182.53, *p* < .0001) were found. However, state and congruency did not significantly interact (F(1, 42) = 1.58, *p* = .22). Thus, Stroop interference did not differ significantly across sessions, however, slowed responses to both congruent and incongruent items were revealed.

With regards to repeated measures analysis of Eriksen's Flanker task, response time performance differed significantly across state (F = (1, 42) = 4.59, *p* = .04) and there were also significant main effects of compatibility (F(1, 42) = 41.69, p < .0001) and distance (F(1, 42) = 74.13, p < .0001). A first order interaction of distance and compatibility was revealed (F(1, 42) = 55.8, p < .0001) but state and compatibility (F(1, 44) = 0.05, *p* = .83), state and distance (F(1, 42) = 0.21, *p* = .65), and state, compatibility and distance (F(1, 42) = 1.48, *p* = .23) did not significantly interact.

From Table 2 it is evident that performance on other tests was also significantly impaired on the hangover day. On the hangover day, significantly fewer words were recalled in the free recall task (t(42), 3.91, p < .0001) and in particular those at the end of the word list (recency) (t(42) = −2.95, p < .0001). In the spatial working memory task, significantly more errors were made on the hangover day (t(42) = 2.26, p = .03). Finally, analysis on the choice reaction time task revealed that response times were significantly longer on the hangover day when compared to the control day (t(42) = 4.02, p < .0001).

Mood assessments are summarized in Table 3. The analysis revealed that, in addition to several individual mood items, both the alertness sum score (*p* < .001) and the tranquility sum score (*p* = .003) were significantly reduced in the alcohol hangover condition.

**Table 3:** Subjective mood ratings. Significant differences (p < .003, after Bonferroni correction) between the hangover and control day are indicated by *.

	Hangover day	Control day	p-Value
Alert/drowsy	3.0 (1.9)	1.2 (1.3)	<0.001*
Contented/discontented	1.9 (1.8)	1.0 (1.4)	<0.001*
Calm/excited	1.4 (1.5)	1.0 (1.5)	0.28
Troubled/tranquil	4.2 (1.8)	4.4 (1.9)	0.50
Strong/feeble	3.0 (1.8)	1.6 (1.5)	<0.001*
Mentally slow/quick witted	2.6 (1.8)	4.1 (1.7)	<0.001*
Muzzy/clear-headed	2.7 (1.8)	4.7 (1.7)	<0.001*
Tense/relaxed	4.0 (2.0)	4.9(1.6)	0.02
Attentive/dreamy	2.9 (2.0)	1.8 (1.7)	0.01
Incompetent/proficient	3.8 (1.8)	4.6 (1.6)	0.03
Happy/sad	1.4 (1.6)	0.9 (1.5)	0.03
Antagonistic/friendly	4.6 (2.0)	5.5 (1.2)	0.01
Interested/bored	1.6 (1.9)	0.9 (1.6)	0.03
Withdrawn/sociable	4.0 (1.9)	6.6 (9.5)	0.08
Depressed/elated	3.8 (1.4)	4.7 (1.5)	<0.001*
Self-centered/outward-going	3.6 (1.6)	4.6 (1.7)	0.01
Well-coordinated/clumsy	3.4 (2.2)	1.6 (1.8)	<0.001*
Lethargic/energy	2.3 (1.9)	4.7 (1.4)	<0.001*
Alertness scale score	40.5 (13.5)	51.3 (10.7)	<0.001*
Tranquility scale score	33.1 (9.5)	40 (12.7)	0.003*

## 4 Discussion

The present study demonstrated significant performance impairment during alcohol hangover on Stroop performance, selective attention, intra- and extra dimensional set shifting, spatial working memory, free recall, and choice reaction time tasks. Reaction times were significantly slower during alcohol hangover and the number of errors was significantly increased, especially in tasks with high difficulty levels.

On the Stroop test, significantly slower responses were found on the hangover day compared to the alcohol-free control day. These results corroborate findings from another study conducted in students by McKinney et al. (2012). However, Stroop interference did not reach significance in the current study. This is likely due to overall slowed responses during a hangover irrespective of congruency. Thus, despite differences in drinking experience (i.e., years of alcohol consumption), it appears that Stroop performance is impaired in both student and non-student samples.

In line with previous research using the Eriksen Flanker Task in a student sample (McKinney & Coyle, 2004), during alcohol hangover response times were slower. However, in this non-student sample, the differences only reached significance on overall response times. As with the Stroop task, overall performance was slowed. Differences in compatibility and distance did not reach significance. The results from the free recall task showed that significantly less words were recalled during an alcohol hangover. This finding corroborates with previous results from studies in student samples (Howland et al., 2010; McKinney & Coyle's, 2004), indicating that free recall is impaired across samples of varying drinking experience and age.

The slower response times observed during an alcohol hangover on the choice reaction time task are in line with previous findings in student samples showing that decision making and motor skills are impaired the morning after a night's drinking (Grange et al., 2016; McKinney & Coyle, 2004). No differences in errors were found between the hangover and control day on the Attentional Set-shifting task. In the spatial working memory task, significantly more errors were made on the hangover test day compared to the control day. A better strategy to complete the task was applied in the control condition than on the hangover test day, however, this did not reach significance. Taken together, the effects on cognitive performance during the hangover state of the current adult working sample were more or less comparable to those observed in student samples. Thus, the impairing effects of alcohol hangover on mood and cognition seen in student samples are equally present in older non-student samples. The implications of this observation are evident. As most adults have a job, these findings support the need for measures to be taken around safety critical working environments, such as transportation, health care, and oil and gas rigs. For example, human errors are responsible for 70% of accidents on oil and gas rigs that can cost up to around £2 billion per accident (Health and Safety Executive, 1999). Alcohol consumption is forbidden during working hours on oil and gas rigs (International Association of Oil and Gas Producers, 2016), but there are no regulations in place that take into account possible next-day hangover effects on work performance. Taking into account the potential negative effects of alcohol hangover on work performance and safety may be beneficial to a wide range of organizations who wish to reduce human error related accidents in the workplace.

Several subjective ratings for individual mood items differed significantly between the hangover and control day Also, the scores on the alertness and tranquility subscales were significantly lower on the hangover day. These findings are similar to those observed in student populations in which various mood ratings usually elevated during the hangover state (Penning et al., 2012; Van Schrojenstein Lantman, Mackus, van de Loo, & Verster, 2017). Alcohol consumption levels of the current sample were relative high, and most participants can be classified as binge drinkers. On the night before the hangover test day participants consumed an average of 15.3 UK units of alcohol. This amount of alcohol is comparable to that consumed in several naturalistic hangover studies in students (Finnigan, Schulze, Smallwood, & Helander, 2005; Hogewoning et al., 2016).

There was no significant difference in hours of sleep between the hangover and the control test days (see Table 1). This finding is in contrast to what is usually observed in student samples in which total sleep time is usually significantly shorter after the evening of alcohol consumption (e.g., Hogewoning et al., 2016; Van Schrojenstein Lantman, Mackus, Roth, & Verster, 2017; Van Schrojenstein Lantman, Roth, Roehrs, & Verster, 2017). A possible explanation for this discrepancy may be found in the days of testing. Students often schedule their social activities (which include alcohol consumption) on week days. At the same time, their obligations with respect to study and class attendance also fall on week days. Given that they have appointments next morning, the time spent drinking is likely to go at the expense of their total sleep time. In contrast, the current sample was tested on free living days (without study, training or work obligations) and these participants got up later, which may explain the absence of significant differences.

Strengths of this study include the fact that it is one of the few hangover studies conducted in a non-student sample, and using a naturalistic study design including real-life drinking levels. The importance of this study lies in the confirmation that in non-student samples of older age comparable performance and mood changes were found as observed in student samples. However, as they fulfill different roles in society, the consequences of these impairments are likely to be different for non-student samples and students. Obligations towards work and family are likely to be different. Whereas students sometimes have the opportunity to skip educational activities or social appointments when hungover, for older adults with jobs absenteeism can have serious consequences. The results may be presenteeism, with reduced productivity and increased risks of accidents or injury.

A limitation of the current study is the variation in testing times. Participation took place from the opening of the premises (between 10.30 a.m. and 12.30.p.m) until 3 p.m. in accordance with public house legal opening hours (Vintners Federation of Ireland, 2018). Diurnal effects have been found to impact cognitive performance and mood. For example, research showed that short term memory is superior early in the morning and deteriorates over the day (Baddeley, Hatter, Scott, & Snashall, 1970). Also, hangover severity varies during the day, and different severity patterns have been identified to characterize drinkers (Verster et al., 2018). Although we aimed to test a participant at approximately the same time of day on the hangover and control day, given the naturalistic design this was not always achieved. Future studies should implement time of day analysis investigation if diurnal effects confound performance while in a hungover state. This can be most easily done in controlled laboratory experiments.

Taken together, the current study in a non-student sample confirms previous findings in student samples that cognitive functioning and mood are significantly impaired during alcohol hangover.

## Declarations

### Authors' contributions

LD, KC, and JV made substantial contributions to conception and design, LD analyzed the data, LD and JV drafted the manuscript, and all authors revised it critically for important intellectual content and approved the final version of the manuscript.

### Declaration of Competing Interests

Over the past three years, Joris Verster has received grants/research support from the Dutch Ministry of Infrastructure and the Environment, Janssen, Nutricia, and Sequential, and acted as a consultant/advisor for Clinilabs, Janssen, Jazz, More Labs, Red Bull, Sen-Jam Pharmaceutical, Toast!, Vital Beverages, and ZBiotics. The other authors have no potential conflicts of interest to disclose.

### Availability of data and materials

The dataset analyzed during the current study is available from the corresponding author on reasonable request.

### Funding

This study was funded by Ulster University.
